# Virtual Geriatric Mental Health Care Offered Where Needed Most: An Evaluation of a Telehealth Consultation Model

**DOI:** 10.1093/geroni/igab046.109

**Published:** 2021-12-17

**Authors:** Julie Filips, Chalise Carlson, Ana Alfaro, Ranak Trevedi, Anita Savell, Christine Gould

**Affiliations:** 1 VAMC, Minneapolis, MN, Minnesota, United States; 2 VA Palo Alto Health Care System, Palo Alto, California, United States; 3 VA Palo Alto Center for Innovation to Implementation, Menlo Park, California, United States; 4 CRH Operations for VISN 23, Minneapolis, Minnesota, United States

## Abstract

Many VA facilities serving large rural populations do not have geriatric mental health specialists available to assist with managing the aging Veteran population’s complex medical and behavioral comorbidities. We applied mixed-methods to evaluate an innovative model utilizing a geriatric psychiatrist who provides cross-facility consultation in a 5-state region. During a 3-month period, the consultant completed 135 consults and 20 e-consults to settings ranging from outpatient to long-term care. Leadership stakeholder and provider interviews highlight the importance of the availability of the consultant, collaboration with local care teams, staff education, person-centered approach, and work ethic/passion. The core challenges that the consultant helps manage include complex comorbidities, medication questions, and dementia with behavioral disturbance. Initial provider survey responses (n = 11) show high satisfaction with services (100%) and strong agreement (80%) that providers could follow through with recommendations. Next steps include replication of this model in other VA facilities.

